# Impacts of caring for a child with the CDKL5 disorder on parental wellbeing and family quality of life

**DOI:** 10.1186/s13023-016-0563-3

**Published:** 2017-01-19

**Authors:** Yuka Mori, Jenny Downs, Kingsley Wong, Barbara Anderson, Amy Epstein, Helen Leonard

**Affiliations:** 1 Telethon Kids Institute, The University of Western Australia, 100 Roberts Road, Subiaco, 6008 Perth, Western Australia; 2 School of Physiotherapy and Exercise Science, Curtin University, Perth, Western Australia

**Keywords:** Parental wellbeing, Family quality of life, CDKL5 disorder, Genetic disorder, Rare disorder

## Abstract

**Background:**

Although research in this area remains sparse, raising a child with some genetic disorders has been shown to adversely impact maternal health and family quality of life. The aim of this study was to investigate such impacts in families with a child with the CDKL5 disorder, a newly recognised genetic disorder causing severe neurodevelopmental impairments and refractory epilepsy.

**Methods:**

Data were sourced from the International CDKL5 Disorder Database to which 192 families with a child with a pathogenic *CDKL5* mutation had provided data by January 2016. The Short Form 12 Health Survey Version 2, yielding a Physical Component Summary and a Mental Component Summary score, was used to measure primary caregiver’s wellbeing. The Beach Center Family Quality of Life Scale was used to measure family quality of life. Linear regression analyses were used to investigate relationships between child and family factors and the various subscale scores.

**Results:**

The median (range) age of the primary caregivers was 37.0 (24.6–63.7) years and of the children was 5.2 (0.2–34.1) years. The mean (SD) physical and mental component scores were 53.7 (8.6) and 41.9 (11.6), respectively. In mothers aged 25–54 years the mean mental but not the physical component score was lower than population norms. After covariate adjustment, caregivers with a tube-fed child had lower mean physical but higher mean mental component scores than those whose child fed orally (coefficient = −4.80 and 6.79; *p* = 0.009 and 0.012, respectively). Child sleep disturbances and financial hardship were negatively associated with the mental component score. The mean (SD) Beach Center Family Quality of Life score was 4.06 (0.66) and those who had used respite services had lower scores than those who had not across the subscales.

**Conclusions:**

Emotional wellbeing was considerably impaired in this caregiver population, and was particularly associated with increased severity of child sleep problems and family financial difficulties. Family quality of life was generally rated lowest in those using respite care extensively, suggesting that these families may be more burdened by daily caregiving.

## Background

The CDKL5 disorder is a recently identified genetic disorder caused by mutations in the X-linked cyclin-dependent kinase-like 5 (*CDKL5*) gene [1]. These mutations were originally identified in children or adults who had been clinically diagnosed with epileptic encephalopathy [2] or the early-onset seizure variant of Rett syndrome (RTT) [3]. However, there has been recent evidence demonstrating some differences in clinical features when compared with typical RTT [4–6]. Epilepsy is almost universal and generally occurs in early infancy with a median age of six weeks ranging from one week to 1.5 years [4, 7] and thus symptoms of this disorder appear very early in life. Sleep difficulties appear to be more common in the CDKL5 disorder than in RTT [4, 6]. Genotype-phenotype relationships have been difficult to study because of the paucity of recurrent mutations and the generally small case series. Our recent study (*n* = 127) which examined early development in this disorder categorised mutation types into four groups according to the effect on protein function and found that compared with those with no functional protein, those with a truncating mutation after amino acid (aa) 781 were more likely to acquire motor and communication skills [8].

Growing evidence suggests that raising a child with a disability greatly impacts the welfare of the primary caregiver, particularly, the mother. Although there is an increasing number of studies examining maternal health [9, 10], research specifically targeting families caring for a child with a genetic disorder remains relatively scarce. While impaired mental health has been reported for those with a child with Down syndrome [11] and RTT [12, 13], and increased stress for those with a child with Prader-Willi syndrome [14], there are no data on the health of parents caring for a child with the CDKL5 disorder.

The concept of family quality of life for those with a child with a developmental disability has developed along with understanding of the crucial role that family plays in raising a child with a disability [15]. Identifying specific challenges for the family is critical to providing an inclusive and multidisciplinary healthcare strategy [16]. The burden of care may impact more greatly on families with a child who have more severe neurodevelopmental impairments accompanied with complex comorbidities and this is typical in many rare disorders. Often, the child’s diagnosis and access to syndrome-specific family support can be considerably delayed, adversely affecting parental wellbeing [17] and possibly also family quality of life.

The aim of this study is to examine primary caregivers’ wellbeing and family quality of life among families with a child living with the CDKL5 disorder, and determine the relationships with a range of factors from child’s characteristics through family circumstances to availability of public resources. This study is the first investigation into family life for those raising a child with the CDKL5 disorder.

## Methods

### Data collection

Prior to 2012 the international Rett syndrome database, InterRett, set up in 2002, and mainly collecting data using electronic data submission [4, 18], also included cases with the CDKL5 disorder. In 2012 the International CDKL5 Disorder Database (ICDD) was established by recontacting those InterRett families in whose child a CDKL5 gene mutation had been identified, and by recruiting new cases in collaboration with the International Foundation for CDKL5 Research (IFCR). Following registration on the IFCR webpage, an ICDD staff member contacts the family, explains the study and provides instructions on how they can complete the questionnaire and provide their genetic test results. The questionnaire comprises two primary components and an epilepsy diary. The first part includes sections that provide a comprehensive picture of the child’s clinical condition from birth to present: perinatal details; primary concerns and diagnosis; developmental milestones; regression; current functional ability, including gross motor, communication and feeding; past and current seizure frequency and medication; sleep difficulties; emotional and social development; stereotypic hand movements; gastrointestinal symptoms; skeletal and muscular health; other comorbidities; hospitalisations; drug treatments; stage of puberty; current body measurements; equipment and intervention use; day care and educational supports; and utilisation of respite care and financial aids. Questions were also asked about the specific forms of respite care used, where formal respite included services provided by public or private organisations and informal respite referred to any service offered by other family members, friends and neighbours. The second part includes sections regarding family demographics, family wellbeing using the Beach Center Family Quality of Life (BCFQOL) Scale, and parental wellbeing using the Short Form 12 Health Survey Version 2 (SF-12v2).

For the current study, data were collected between September 2012 and January 2016. Of those with a confirmed pathogenic mutation, 192 of 209 provided questionnaire data. One hundred and fifty-eight families (82.2% of 192) were included in the parental health analysis excluding 34 of those with three or more missing items. Of those, 141 (89.2%) questionnaires were completed by the biological mother, 15 (9.5%) by the biological father, and one (0.6%) each by a foster mother and a grandparent. In the family quality of life analysis, 149 families with fewer than three missing items (77.6% of 192) were included. The questionnaires were filled out by 131 (87.9%) biological mothers, 16 (10.7%) biological fathers, one foster mother (0.7%) and one grandparent (0.7%).

### Measurements

#### Child’s mutation group

There was considerable variability in mutations in the CDKL5 gene with 149 unique and only 18 recurrent mutations. Hence, every mutation was categorised into one of five groups according to the subsequent protein function: No functional protein; Missense/in-frame mutations within kinase domain; Truncations between aa172 and aa781; Truncations after aa781; and mutations that could not be grouped [8].

#### Child’s functional abilities

For those aged 1.5 years or over, abilities to sit on the floor for ten seconds and to walk ten steps were categorised as needing no assistance, needing some support or unable. Also in those aged 1.5 years or over, ability to communicate was categorised as using no or simple communication methods (eg, body language and facial expressions), complex gestures and vocalisation (eg, selects or rejects a photo of object, points at an object or returns an unwanted item) or using sign or spoken language (eg, single words and sentences). Children or adults who were totally dependent on enteral feeding were excluded from analyses relating to dietary concerns.

#### Child’s sleep

The Sleep Disturbance Scale for Children (SDSC) has been validated in children with disability as well as healthy children [19]. It contains 26 items rated on a five-point Likert scale [19]. Four items were not included in the questionnaire as they were not appropriate to this population (e.g. the child sleep walks). The average score of the remaining 22 items was calculated with a possible range of one to five among those with no more than two missing items. Higher scores indicate poorer quality of the child’s sleep and data were stratified into quartile groups.

#### Child’s hospitalisations

A hospital admission rate was calculated by dividing the total number of reported hospitalisations by the child’s age. Data were then stratified into quartile groups.

#### Primary caregiver wellbeing

The SF-12v2 is a valid and reliable instrument of assessing self-reported health and wellbeing [20]. It comprises twelve items that form two scales, the Physical Component Summary (PCS) and Mental Component Summary (MCS), and eight subscales, including Physical Functioning, Role Physical, Bodily Pain, General Health, Vitality, Social Functioning, Role Emotional and Mental Health [20]. The score is calculated based on a norm-referencing method using the US general norms collected in 1998 with a mean score of 50 and standard deviation (SD) of 10. Higher scores indicate better health status. Overall, 149 individuals answered all items, and seven and two families had one and two missing items, respectively. For each of eight subscales, a mean value among those without any missing data was applied to best estimate when the subscale score calculation resulted in failure due to the missing items.

#### Family quality of life

The BCFQOL Scale assesses family quality of life, specifically, for those raising children with a disability [21, 22]. The instrument, originally developed in the US, has been used in other countries including Australia and Spain [21]. It comprises 25 items with five subscales: Family interaction; Parenting; Emotional wellbeing; Physical/Material wellbeing; and Disability-related support [22]. The average of total item scores is obtained for each factor, with a possible value of one to five (three and four are rated when a family are neither satisfied or dissatisfied and satisfied with an item, respectively). Higher scores indicate better family quality of life. In total, 130 families responded to every item, and 16 and three individuals had one and two missing items, respectively.

#### Analysis

The PCS and MCS scores of the SF-12v2, and five factor scores of the BCFQOL Scale were the dependent variables. Univariate linear regression analyses were performed to investigate their crude relationships with the independent variables including family aspects such as parent’s age and employment status, child’s characteristics such as current functional ability and SDSC scores, and use of social supports such as respite care services. Multivariate linear regression analyses aimed to examine effects of the child’s clinical symptoms and socio-environmental factors on the SF-12v2 and BCFQOL scores and included adjustment for potential confounders, including socio-demographic factors, family composition and child’s age. These variables comprised parent’s age, highest qualification and work status, as well as child’s age, birth order, number of siblings, and country of birth. Age was a continuous variable and qualification and country of birth were binary variables (i.e. university degree or lower, North America or others, respectively) in the models. Multivariate linear regression models for subscales of the BCFQOL Scale included the PCS and MCS as continuous variables as well as all the abovementioned adjustors because the SF-12v2 and BCFQOL scale were scored by the same family member. Differences in mean PCS and MCS scores between 141 biological mothers and the US female norms were assessed using t-tests. The statistical package Stata 14 (StataCorp LP, College Station, TX) was used for all the analyses.

## Results

Table 1 shows the child and family characteristics if the child had a confirmed pathogenic mutation in the *CDKL5* gene, regardless of whether they fulfilled inclusion criteria for the analyses. At ascertainment, biological parents ranged in age from 24.6 to 63.7 years with a median of 38.5 years. Approximately 60% (59.4%) had university degrees, whereas nearly half (47.5%) were full-time homemakers or unemployed. The child’s median age was 5.2 years, varying from 0.2 to 34.1 years, and the majority (85.4%) were females. Over half of the children (52.4%) were born in the US, followed by the United Kingdom (UK) and Australia (both 7.3%). More than three quarters (77.2%) had siblings and nearly half (46.3%) were the first child for the parents. Each of the following mutation groups (no functional protein, missense or in-frame mutations within kinase domain, and truncations from aa172 to aa781) accounted for between a quarter and a third of the cases.

**Table 1:** Family demographics and the child’s characteristics in the database (*n*=192)

Family factors	
Parental age (*n*=160) mean (SD), years	38.2 (7.0)
*n* (%)	
Under 30 years	14 (8.8)
30 to 40 years	84 (52.5)
40 years or over	62 (38.8)
Parental qualification (*n*=160), *n* (%)	
11 or 12 years of school	34 (21.3)
TAFE/technical certificate	31 (19.4)
University degree	95 (59.4)
Parental work status (*n*=160), *n* (%)	
Full-time homemaker/unemployment	76 (47.5)
Part-time employment	39 (24.4)
Full-time employment	45 (28.1)
Number of siblings (*n*=162), *n* (%)	
0	37 (22.8)
1	67 (41.4)
2 or more	58 (35.8)
Birth order (*n*=162), *n* (%)	
The first child	75 (46.3)
Laterborn	87 (53.7)
Child factors	
Child’s age (*n*=192) mean (SD), years	7.1 (6.1)
*n* (%)	
Under 1.5 years	30 (15.6)
1.5 to 7 years	86 (44.8)
7 to 13 years	48 (25.0)
13 years	28 (14.6)
Child’s gender (*n*=192), *n* (%)	
Female	164 (85.4)
Male	28 (14.6)
Child’s country of birth (*n*=191), *n* (%)	
North America	105 (55.0)
The United Kingdom	14 (7.3)
Australia and New Zealand	16 (8.4)
Western Europe	38 (19.9)
Others	18 (9.4)
Mutation group (*n*=192), *n* (%)	
No functional protein	55 (28.7)
Missense/In-frame mutations within kinase domain	48 (25.0)
Truncations from aa172 to aa781	58 (30.2)
Truncations after aa781	16 (8.3)
Mutations not grouped	15 (7.8)
Feeding pattern (*n*=171), *n* (%)	
Oral intake only	128 (74.9)
Combination use^#2^	16 (9.4)
Enteral nutrition only^#3^	27 (15.8)
Dietary concerns (*n*=139)^#4^, *n* (%)	
None	66 (47.5)
Occasional	47 (33.8)
Frequent/constant	26 (18.7)
Floor sitting (*n*=144)^#5^, *n* (%)	
No assistance	89 (61.8)
Some assistance	14 (9.7)
Mximal assistance/unable	41 (28.5)
Taking 10 steps forward (*n*=141)^#5^, *n* (%)	
No assistance	30 (21.3)
Some assistance	20 (14.2)
Mximal assistance/unable	91 (64.5)
Eye contact (*n*=158), *n* (%)	
No	85 (53.8)
Yes	73 (46.2)
Communication (*n*=143)^#5^, *n* (%)	
No/Simple communication	53 (37.1)
Complex gestures/vocalisation	62 (43.4)
Sign/Spoken language	28 (19.6)
Seizure frequency (*n*=160), *n* (%)	
Absent	14 (8.8)
Yearly/Monthly	16 (10.0)
Weekly	27 (16.9)
1 to 5 times per day	74 (46.3)
At least 5 times per day	29 (18.1)
Sleep difficulties (average SDSC score) (*n*=155), *n* (%)	
1 to 1.50 (1^st^ quartile)	38 (24.5)
1.50 to 1.81 (2^nd^ quartile)	39 (25.2)
1.81 to 2.17 (3^rd^ quartile)	40 (25.8)
2.17 to 5 (4^th^ quartile)	38 (24.5)
Hospitalisation rate (*n*=156), per 1 person-year, *n* (%)	
0 to 0.38 (1^st^ quartile)	39 (25.0)
0.38 to 0.86 (2^nd^ quartile)	39 (25.0)
0.86 to 2.14 (3^rd^ quartile)	39 (25.0)
2.14 to 13.70 (4^th^ quartile)	39 (25.0)
Socio-environmental factors	
Respite care (*n*=153), *n*(%)	
None	41 (26.8)
Formal respite care only	21 (13.7)
Informal respite care only	41 (26.8)
Both formal and informal respite care	50 (32.7)
Financial difficulty (*n*=147), *n* (%)	
No	83 (56.5)
Yes	64 (43.5)

^#1^ North America includes the United States (100) and Canada (5). Australia and New Zealand contains Australia (14) and New Zealand (2). Western Europe includes Germany (12), France (7), the Netherlands (6), Ireland (3), Spain (2), Italy (2), Belgium (1), Denmark (1), Finland (1), Norway (1), Sweden (1) and Luxemburg (1). Others comprises Brazil (3), India (2), Russia (2), Hungary (2), Bulgaria (1), Poland (1), Slovenia (1), Israel (1), China (1), Singapore (1), Mexico (1), Perto Rico (1) and Argentina (1).

^#2^ 14 used a gastrostomy tube, 2 used a nasogastric tube

^#3^ 25 used a gastrostomy tube, 2 used a nasogastric tube

^#4^ Among children who intook food orally with or without combination use of external nutrition

^#5^ Excludes child aged younger than 1.5 years

Over 60% (61.8%) of those aged 1.5 years or more were able to sit on the floor without any assistance, whereas only 21.3% were able to walk independently. Less than one fifth (19.6%) were able to communicate using sign or spoken language and approximately a half (53.8%) avoided eye contact. Almost two thirds (64.1%) currently had epileptic seizures on a daily basis. Almost one sixth (15.8%) of the children were dependent on enteral nutrition and the majority (25 of 27) of this group had a gastrostomy. More than half (52.5%) of mothers of those whose children were orally fed, including combination use with enteral feeding, were concerned about the amount of food and/or liquid their child consumed. Almost three quarters (73.2%) of the families had utilised a form of respite care services and with nearly a third (32.7%) receiving both the informal and public services. Nearly half (43.5%) had experienced financial hardship to meet their child’s healthcare needs.

### Primary caregiver wellbeing

The mean PCS score was 53.7 (SD 8.6) ranging from 28.0 to 72.7. The univariate analysis identified that the child’s country of birth had the greatest variability in the scores such that mothers of children who were born in Australia and New Zealand, and Western European countries had lower scores than those of children who were born in North America (coefficients = −10.98 and −9.21; *p* <0.001 and *p* <0.001, respectively) (Table 2). Those whose child was dependent on enteral nutrition had considerably poorer physical health (mean PCS score 49.6) compared with those whose child fed orally (mean PCS score 54.3; coefficient = −4.72; *p* = 0.013), as had part-time workers (mean PCS score 50.4) compared with full-time homemakers (mean PCS score 55.1; coefficient = −4.69; *p* = 0.006). The negative effect of enteral feeding remained significant in the multivariate analysis (coefficient = −4.51; *p* = 0.015) (Table 3). There was slight improvement in primary caregiver’s physical health for those who used both forms of respite care (coefficient = 1.00; *p* = 0.603).

**Table 2:** Univariate analysis for PCS and MCS of the SF-12v2 (*n* = 158)

Measure	Number of observations	PCS		MCS	
		Mean (SD) 53.7 (8.6)	Co-efficient	Mean (SD) 41.9 (11.6)	Co-efficient
Parent’s age	152				
Under 30 years	12	53.5 (5.9)	Reference	35.9 (15.7)	Reference
30 to 40 years	80	54.7 (8.7)	1.23	41.9 (11.3)	5.97
40 years or over	60	52.9 (8.7)	−0.59	43.3 (11.3)	7.37**
Parent’s qualification	147				
11 or 12 years of school	27	53.2 (7.9)	Reference	41.8 (13.2)	Reference
TAFE/Technical certificate	29	53.6 (9.3)	0.40	41.2 (9.0)	−0.57
University degree	91	54.4 (8.4)	1.14	42.0 (12.1)	−0.23
Maternal work status	147				
Full-time homemaker	64	55.1 (8.1)	Reference	42.7 (11.3)	Reference
Part-time employment	37	50.4 (9.4)	−4.69**	42.4 (11.9)	−0.32
Full-time employment	46	55.7 (7.5)	0.57	40.8 (11.2)	−1.86
Number of siblings	156				
0	36	54.3 (8.1)	Reference	37.4 (13.8)	Reference
1	64	54.4 (7.5)	0.07	41.9 (11.3)	4.47*
2 or more	56	52.9 (9.9)	−1.41	45.0 (9.6)	7.56**
Child order	156				
The first child	72	54.4 (7.9)	Reference	40.2 (11.7)	Reference
Laterborn	84	53.3 (9.1)	−1.02	43.5 (11.4)	3.24*
Child’s age	158				
Under 1.5 years	24	53.3 (10.0)	Reference	39.8 (13.2)	Reference
1.5 to 7 years	69	54.6 (7.6)	1.31	42.4 (12.4)	2.63
7 to 13 years	40	52.7 (9.8)	−0.63	43.7 (9.7)	3.94
13 years or over	25	53.0 (8.0)	−0.29	39.2 (10.7)	−0.55
Child’s gender	158				
Female	136	53.9 (8.3)	Reference	41.8 (11.6)	Reference
Male	22	52.3 (10.4)	−1.60	41.9 (12.1)	0.10
Child’s country of birth	158				
North America	90	56.9 (5.9)	Reference	41.5 (12.1)	Reference
The United Kingdom	12	54.2 (10.9)	−2.63	41.1 (8.4)	−0.40
Australia and New Zealand	14	45.9 (7.4)	−10.98**	43.6 (12.5)	2.07
Western Europe	29	47.6 (10.0)	−9.21**	41.2 (12.2)	−0.30
Others	13	52.9 (8.2)	−3.93*	44.5 (9.7)	3.02
Mutation group	158				
No functional protein	50	53.5 (8.0)	Reference	43.0 (12.0)	Reference
Missense/In-frame mutations	41	52.5 (9.9)	−0.97	39.5 (10.8)	−3.49
Truncations aa172 to aa781	44	53.7 (8.7)	0.23	42.0 (11.5)	−0.98
Truncations after aa781	12	53.6 (7.4)	0.15	46.2 (9.1)	3.21
Mutations not grouped	11	58.5 (6.5)	5.05*	39.5 (15.1)	−3.49
Feeding pattern	153				
Oral intake only	114	54.3 (8.1)	Reference	41.0 (12.2)	Reference
Combination use	14	55.0 (6.8)	0.74	39.7 (9.4)	−1.32
Enteral nutrition only	25	49.6 (11.0)	−4.72**	47.4 (9.3)	6.37**
Dietary concerns	123				
None	62	55.9 (7.3)	Reference	41.9 (11.7)	Reference
Occasional	39	51.8 (7.4)	−4.14**	39.9 (12.6)	−2.00
Frequent or constant	22	55.7 (8.0)	−0.25	42.5 (10.3)	0.67
Floor sitting	129				
No assistance	83	54.3 (7.6)	Reference	41.7 (11.7)	Reference
Some assistance	13	54.5 (7.8)	0.20	42.0 (12.5)	0.27
Maximal assistance/unable	33	51.3 (10.3)	−3.02*	43.9 (10.6)	2.25
Taking 10 steps forward	126				
No assistance	28	54.8 (7.2)	Reference	41.6 (12.6)	Reference
Some assistance	19	57.3 (7.2)	2.52	40.6 (14.3)	−0.95
Maximal assistance/unable	79	52.4 (8.9)	−2.37	43.2 (10.4)	1.63
Eye contact	148				
No	78	53.0 (9.6)	Reference	41.0 (12.0)	Reference
Yes	70	54.6 (7.3)	1.57	42.5 (11.7)	1.54
Communication	129				
No/Simple method	44	53.0 (9.0)	Reference	44.1 (9.6)	Reference
Complex gestures/vocalisation	58	53.5 (8.5)	0.52	41.2 (12.4)	−2.91
Sign/Spoken language	27	55.0 (7.5)	1.98	42.1 (12.0)	−2.02
Seizure frequency	146				
Absent	12	53.7 (7.5)	Reference	43.2 (11.3)	Reference
Yearly/Monthly	15	53.4 (9.8)	−0.33	41.3 (13.1)	−1.82
Weekly	26	54.5 (6.7)	0.84	40.5 (10.7)	−2.71
1 to 5 times a day	69	53.4 (8.9)	−0.28	41.1 (12.7)	−2.09
At least 5 times a day	24	54.2 (9.8)	0.49	45.6 (8.5)	2.47
Sleep difficulties	144				
1.00 to 1.50 (1^st^ quartile)	36	54.5 (8.3)	Reference	45.2 (11.3)	Reference
1.50 to 1.81 (2^nd^ quartile)	38	54.1 (7.7)	−0.46	42.7 (10.8)	−2.58
1.81 to 2.17 (3^rd^ quartile)	35	54.6 (7.5)	0.11	42.6 (11.2)	−2.63
2.17 to 5.00 (4^th^ quartile)	35	52.5 (10.1)	−2.00	38.2 (12.1)	−7.06**
Hospitalisation rate	146				
0 to 0.38 (1^st^ quartile)	34	52.9 (7.8)	Reference	42.5 (10.2)	Reference
0.38 to 0.86 (2^nd^ quartile)	38	54.6 (8.3)	1.66	43.0 (12.2)	0.50
0.86 to 2.14 (3^rd^ quartile)	36	53.1 (8.4)	0.23	39.9 (10.3)	−2.60
2.14 to 13.70 (4^th^ quartile)	38	54.0 (9.7)	1.11	43.7 (12.2)	1.19
Respite care	148				
None	36	54.4 (8.3)	Reference	42.7 (11.8)	Reference
Formal only	21	52.0 (10.1)	−2.41	44.9 (8.3)	2.21
Informal only	41	54.1 (8.6)	−0.26	41.1 (13.9)	−1.56
Both formal and informal	50	53.2 (8.4)	−1.25	40.8 (10.4)	−1.85
Financial difficulty	144				
No	83	53.9 (8.5)	Reference	44.0 (11.2)	Reference
Yes	61	52.9 (8.9)	−0.97	39.1 (11.6)	−4.89**

*p <0.10 **p <0.05

**Table 3:** Multivariate analysis for PCS and MCS of the SF-12v2 and coefficients^#1^

Measure		PCS	MCS
		Coefficient (95% CI)	Coefficient (95% CI)
Feeding pattern	Oral intake only	Reference	Reference
	Combination use	−1.01 (−5.63, 3.60)	−1.49 (−8.36, 5.37)
	Enteral nutrition only	−4.51** (−8.12, −0.89)	6.67** (1.29 to 12.05)
Dietary concerns	None	Reference	Reference
	Occasional	−4.98** (−8.11, −1.86)	−0.88 (−5.97, 4.20)
	Frequent or constant	−1.81 (−5.51, 1.88)	0.30 (−5.72, 6.32)
Floor sitting	No assistance	Reference	Reference
	Some assistance	0.21 (−4.01, 4.42)	−1.59 (−8.14, 4.96)
	Maximal assistance/unable	−2.27 (−5.44, 0.90)	1.03 (−3.89, 5.96)
Taking 10 steps forward	No assistance	Reference	Reference
	Some assistance	1.20 (−3.52, 5.91)	−1.23 (−8.59, 6.14)
	Maximal assistance/unable	−1.85 (−5.34, 1.64)	0.15 (−5.30, 5.61)
Eye contact	No	Reference	Reference
	Yes	1.04 (−1.67, 3.75)	1.77 (−2.46, 6.00)
Communication	No/Simple method	Reference	Reference
	Complex gestures/vocalisation	0.45 (−2.58, 3.48)	−0.85 (−5.42, 3.72)
	Sign/Spoken language	1.03 (−3.00, 5.06)	0.14 (−5.93, 6.21)
Seizure frequency	Absent	Reference	Reference
	Yearly/Monthly	−0.55 (−6.78, 5.68)	−2.82 (−12.11, 6.46)
	Weekly	0.42 (−5.41, 6.25)	−3.42 (−12.11, 5.27)
	1 to 5 times a day	−0.76 (−5.88, 4.35)	−1.90 (−9.52, 5.73)
	At least 5 times a day	−0.17 (−6.07, 5.72)	0.09 (−8.69, 8.88)
Sleep difficulties	1.00 to 1.50 (1^st^ quartile)	Reference	Reference
	1.50 to 1.81 (2^nd^ quartile)	0.37 (−3.31, 4.04)	−2.58 (−8.11, 2.95)
	1.81 to 2.17 (3^rd^ quartile)	2.12 (−1.65, 5.89)	−2.57 (−8.25, 3.10)
	2.17 to 5.00 (4^th^ quartile)	1.02 (−2.88, 4.93)	−8.04** (−13.91, −2.16)
Hospitalisation rate	0 to 0.38 (1^st^ quartile)	Reference	Reference
	0.38 to 0.86 (2^nd^ quartile)	−0.60 (−4.49, 3.30)	−1.13 (−7.01, 4.73)
	0.86 to 2.14 (3^rd^ quartile)	−2.27 (−6.42, 1.87)	−4.22 (−10.47, 2.03)
	2.14 to 13.70 (4^th^ quartile)	−1.15 (−5.31, 3.00)	−0.88 (−7.15, 5.38)
Respite care	None	Reference	Reference
	Formal only	0.08 (−4.63, 4.79)	1.54 (−5.63, 8.69)
	Informal only	−0.80 (−4.57, 2.97)	−1.54 (−7.26, 4.18)
	Both formal and informal	1.00 (−2.79, 4.78)	−2.73 (−8.48, 3.02)
Financial difficulty	No	Reference	Reference
	Yes	−1.76 (−4.43, 0.92)	−4.75** (−8.78, −0.71)

^#1^Adjusted for parent’s age (a continuous variable), parent’s qualification (a binary variable; university degree or lower), parent’s work status, number of siblings, the first child, child’s age (a continuous variable) and child’s country of birth (a binary variable; North America or others)

**p <0.05

The mean MCS score was 41.9 (SD 11.6) ranging from 9.3 to 63.2. The number of children in the family, followed by the parent’s age, had the two greatest impacts in the univariate regression analysis (Table 2). Mothers with an only child having the disorder reported the poorest mental wellbeing with a mean of 37.4, compared with having two, and three or more children (coefficient = −4.47 and −7.56; *p* = 0.061 and 0.002, respectively). MCS scores rose with increasing parent’s age, with the mean score of 41.9 for those aged 30 to 40 years and 43.3 for those aged 40 years or more, compared with that of 35.9 in those aged younger than 30 years (*p* = 0.101 and 0.048, respectively). Child factors also impacted greatly on the MCS scores. Severity of the child’s sleep disturbances associated negatively with a mean of 38.2 in the highest quartile (i.e. the greatest difficulty) to 45.2 for the lowest (i.e. the least difficulty) (*p* = 0.010). Mothers of children who were totally dependent on enteral nutrition had the highest MCS with a mean of 47.4, significantly higher than those whose children were totally orally fed (*p* = 0.013). Experiencing financial hardship adversely affected mental health (coefficient = −4.89; *p* = 0.011). The multivariate analysis identified that the child’s sleep disturbances had the largest negative effect on MCS scores (coefficient = −8.04; *p* = 0.008, the highest vs. lowest quartile) (Table 3). The impact of the child’s feeding pattern and financial difficulty experiences remained considerable. Utilisation of respite care services did not appear to be associated with improvement in primary caregiver’s emotional health.

The mean PCS score of 141 of biological mothers was higher overall at 54.0 (SD 8.5), compared with 48.5 (SD 9.9) for the US female norms (*p* <0.001), but for those aged 25 to 34 years the increase was minimal at 1.4 points (95%CI −1.29, 4.13). On the other hand, the mean MCS score for CDKL5 mothers at 41.5 (SD 11.5), was 6.90 points (95%CI −8.57, −5.23) lower that of the US female norms (*p* <0.001). The difference was virtually consistent across the age groups.

### Family quality of life

Family quality of life was generally rated as satisfactory with an overall score of 4.06 (SD 0.66). Among the subscales, emotional wellbeing scores were lowest with a mean of 3.50 (SD 0.97) and physical/material wellbeing score were the highest with a mean of 4.32 (SD 0.70) (Table 4). The univariate analysis demonstrated that the child’s country of birth profoundly influenced the BCFQOL scores, in which those from Western Europe except the UK had the poorest family quality of life across subscales. Use of respite services was associated adversely with every subscale, with lower BCFQOL scores in family interaction, parenting, emotional wellbeing, physical/material wellbeing and disability-related support observed for those who had received both forms of the services compared to non-users (coefficient = −0.50, −0.35, −0.24, −0.34 and −0.44; *p* = 0.001, 0.028, 0.268, 0.027 and 0.011, respectively). There was a negative correlation with the SDSC scores such that the families of the children with the greatest sleep difficulties had the lowest scores with all BCFQOL subscales, with the coefficients of −0.34 for family interaction, −0.34 for parenting, −0.57 for emotional wellbeing, −0.41 for physical/material wellbeing and −0.39 for disability-related support compared to the least sleep disturbances (*p* = 0.054, 0.057, 0.020, 0.015 and 0.040, respectively). Mothers aged 40 years or more reported considerably poorer quality of disability-related support, family interaction and parenting, compared with those aged under 30 years (coefficients = −0.71, −0.45 and −0.34; *p* = 0.005, 0.056 and 0.159, respectively). The multivariate model identified that those with the most extensive utilisation of respite care had the poorest family quality of life with emotional wellbeing subscale scores being the lowest for those who used formal respite care in comparison with those who had never received such services (Table 5). Particularly, the effects on family interaction, emotional wellbeing, physical/material wellbeing and disability-related support were considerable (coefficients = −0.39, −0.36, −0.32 and −0.39; *p* = 0.027, 0.127, 0.048 and 0.044, respectively). The impact of child’s sleep difficulty was attenuated after adjusting for PCS and MCS scores.

**Table 4:** Univariate analysis for the BCFQOL Scale and coefficients (*n* = 149)

Measure	Number of observations	Family interaction	Parenting	Emotional wellbeing	Physical/Material wellbeing	Disability-related support
		Mean (SD) 4.22 (0.71)	Mean (SD) 4.15 (0.72)	Mean (SD) 3.50 (0.97)	Mean (SD) 4.32 (0.70)	Mean (SD) 4.13 (0.77)
Parent’s age	143					
Under 30 years	11	Reference	Reference	Reference	Reference	Reference
30 to 40 years	75	−0.18	−0.13	0.07	0.07	−0.51**
40 years or over	57	−0.45*	−0.34	0.06	−0.06	−0.71**
Parent’s qualification	137					
11 or 12 years of school	24	Reference	Reference	Reference	Reference	Reference
TAFE/Technical certificate	28	0.18	0.10	0.05	0.36*	−0.11
University degree	85	0.13	0.07	0.07	0.28*	−0.11
Parent’s work status	138					
Full-time homemaker	58	Reference	Reference	Reference	Reference	Reference
Part-time employment	34	−0.21	−0.32**	0.18	−0.19	−0.24
Full-time employment	46	0.03	−0.06	0.29	−0.03	−0.24
Number of siblings	147					
0	29	Reference	Reference	Reference	Reference	Reference
1	64	−0.10	0.09	−0.17	0.13	−0.03
2 or more	54	0.04	0.20	−0.10	0.25	0.11
Child order	147					
The first child	63	Reference	Reference	Reference	Reference	Reference
Laterborn	84	0.00	0.10	0.06	0.17	0.05
Child’s age	149					
Under 1.5 years	21	Reference	Reference	Reference	Reference	Reference
1.5 to 7 years	67	0.06	−0.11	−0.32	−0.11	0.09
7 to 13 years	38	0.00	−0.12	−0.35	−0.11	0.05
13 years or over	23	−0.21	−0.34	−0.28	−0.36*	−0.09
Child’s gender	149					
Female	128	Reference	Reference	Reference	Reference	Reference
Male	21	0.13	0.07	0.00	0.01	−0.12
Child’s country of birth	149					
North America	87	Reference	Reference	Reference	Reference	Reference
The United Kingdom	12	−0.02	−0.09	−0.05	−0.20	−0.25
Australia and New Zealand	13	−0.29	−0.48**	−0.21	−0.45**	−0.19
Western Europe	26	−0.51**	−0.52**	−0.43*	−0.46**	−0.61**
Others	11	−0.01	−0.18	−0.30	−0.32	−0.22
Mutation group	149					
No functional protein	46	Reference	Reference	Reference	Reference	Reference
Missense/In-frame mutations	36	0.10	0.03	−0.16	0.01	−0.16
Truncations aa172 to aa781	45	0.05	−0.06	−0.16	0.02	−0.08
Truncations after aa781	12	0.07	0.06	0.11	0.16	0.06
Mutations not grouped	10	−0.01	−0.17	−0.06	−0.13	−0.31
Feeding pattern	144					
Oral intake only	107	Reference	Reference	Reference	Reference	Reference
Combination use	14	−0.13	−0.16	−0.04	−0.34*	−0.10
Enteral nutrition only	23	0.21	0.15	0.15	−0.02	0.06
Dietary concerns	117					
None	57	Reference	Reference	Reference	Reference	Reference
Occasional	38	−0.22	−0.34**	−0.40*	−0.40**	−0.32**
Frequent or constant	22	0.18	−0.20	−0.02	−0.14	−0.20
Floor sitting	123					
No assistance	77	Reference	Reference	Reference	Reference	Reference
Some assistance	13	0.07	0.07	0.29	−0.04	0.22
Maximal assistance/unable	33	0.17	0.19	0.15	0.06	0.15
Taking 10 steps forward	20					
No assistance	26	Reference	Reference	Reference	Reference	Reference
Some assistance	17	0.15	−0.05	−0.03	−0.07	−0.04
Maximal assistance/unable	77	0.16	0.01	0.06	−0.02	0.01
Eye contact	139					
No	71	Reference	Reference	Reference	Reference	Reference
Yes	68	−0.10	−0.11	−0.19	−0.09	−0.05
Communication	123					
No/Simple method	42	Reference	Reference	Reference	Reference	Reference
Complex gestures/vocalisation	57	−0.16	−0.18	−0.38*	−0.16	−0.24
Sign/Spoken language	24	0.06	0.10	−0.02	0.19	0.18
Seizure frequency	138					
Absent	12	Reference	Reference	Reference	Reference	Reference
Yearly/Monthly	15	−0.28	−0.15	−0.07	−0.25	0.12
Weekly	25	−0.29	−0.03	0.14	−0.19	−0.03
1 to 5 times a day	62	−0.15	−0.00	0.11	−0.09	0.09
At least 5 times a day	24	−0.11	0.14	0.39	−0.06	0.22
Sleep difficulties	136					
1.00 to 1.50 (1^st^ quartile)	34	Reference	Reference	Reference	Reference	Reference
1.50 to 1.81 (2^nd^ quartile)	37	−0.24	−0.23	−0.42*	−0.33**	−0.37**
1.81 to 2.17 (3^rd^ quartile)	32	−0.19	−0.20	−0.34	−0.07	−0.14
2.17 to 5.00 (4^th^ quartile)	33	−0.34*	−0.34*	−0.57**	−0.41**	−0.39**
Hospitalisation rate	139					
0 to 0.38 (1^st^ quartile)	31	Reference	Reference	Reference	Reference	Reference
0.38 to 0.86 (2^nd^ quartile)	36	0.19	0.17	0.13	0.15	0.25
0.86 to 2.14 (3^rd^ quartile)	35	0.01	−0.09	−0.42	−0.07	0.15
2.14 to 13.70 (4^th^ quartile)	37	0.20	0.35**	0.06	0.07	0.16
Respite care	140					
None	37	Reference	Reference	Reference	Reference	Reference
Formal only	21	−0.27	−0.18	−0.56**	−0.16	−0.15
Informal only	35	−0.12	−0.19	−0.37	−0.27*	−0.31*
Both formal and informal	47	−0.50**	−0.35**	−0.24	−0.34**	−0.44**
Financial difficulty	137					
No	76	Reference	Reference	Reference	Reference	Reference
Yes	61	0.03	−0.09	−0.57**	−0.22*	−0.09

*p < 0.10 **p < 0.05

**Table 5:** Multivariate analysis for the BCFQOL Scale and coefficients^#1^

Measure		Family interaction	Parenting	Emotional wellbeing	Physical/Material wellbeing	Disability-related support
Feeding pattern	Oral intake only	Reference	Reference	Reference	Reference	Reference
	Combination use	−0.12	−0.16	0.13	−0.16	0.13
	Enteral nutrition only	0.07	0.02	-0.06	-0.03	-0.06
Dietary concerns	None	Reference	Reference	Reference	Reference	Reference
	Occasional	−0.15	−0.25	−0.08	−0.25	−0.19
	Frequent or constant	−0.29	−0.29	0.19	−0.19	−0.26
Floor sitting	No assistance	Reference	Reference	Reference	Reference	Reference
	Some assistance	−0.02	0.05	0.42	−0.10	0.17
	Maximal assistance/Unable	0.17	0.16	0.19	0.14	0.20
Taking 10 steps forward	No assistance	Reference	Reference	Reference	Reference	Reference
	Some assistance	0.21	−0.03	0.13	0.02	0.09
	Maximal assistance/Unable	0.18	0.08	0.25	0.09	0.07
Eye contact	No	Reference	Reference	Reference	Reference	Reference
	Yes	−0.16	−0.18	−0.19	−0.12	−0.05
Communication	No/Simple method	Reference	Reference	Reference	Reference	Reference
	Complex gestures/vocalisation	−0.12	−0.11	−0.33*	−0.11	−0.15
	Sign/Spoken language	−0.02	−0.02	−0.31	0.03	0.06
Seizure frequency	Absent	Reference	Reference	Reference	Reference	Reference
	Yearly/Monthly	−0.28	−0.06	0.40	−0.15	0.26
	Weekly	−0.12	0.13	0.57*	−0.05	0.26
	1 to 5 times a day	−0.25	−0.06	0.13	−0.06	0.18
	At least 5 times a day	−0.07	0.14	0.53	0.06	0.35
Sleep difficulties	1.00 to 1.50 (1^st^ quartile)	Reference	Reference	Reference	Reference	Reference
	1.50 to 1.81 (2^nd^ quartile)	−0.10	−0.08	−0.28	−0.09	−0.28
	1.81 to 2.17 (3^rd^ quartile)	−0.06	−0.03	−0.26	0.08	−0.10
	2.17 to 5.00 (4^th^ quartile)	−0.15	−0.09	−0.26	−0.16	−0.23
Hospitali-sation rate	0 to 0.38 (1^st^ quartile)	Reference	Reference	Reference	Reference	Reference
	0.38 to 0.86 (2^nd^ quartile)	0.14	0.07	0.09	−0.01	0.21
	0.86 to 2.14 (3^rd^ quartile)	−0.07	−0.18	−0.37	−0.22	0.08
	2.14 to 13.70 (4^th^ quartile)	0.10	0.24	0.09	0.03	0.13
Respite care	None	Reference	Reference	Reference	Reference	Reference
	Formal only	−0.29	−0.18	−0.72**	−0.07	−0.01
	Informal only	−0.06	−0.20	−0.47**	−0.23	−0.28
	Both formal and informal	−0.39**	−0.26	−0.36	−0.32**	−0.39**
Financial difficulty	No	Reference	Reference	Reference	Reference	Reference
	Yes	0.11	0.04	−0.27	0.03	0.02

^#1^Adjusted for parent’s age (a continuous variable), parent’s qualification (a binary variable; university degree or lower), parent’s work status, number of siblings, the first child, child’s age (a continuous variable), child’s country of birth (a binary variable; North America or others) and the scores of PCS and MCS (continuous variables)

*p <0.10 **p <0.05

## Discussion

Caregivers of children with the CDKL5 disorder experienced considerable emotional burden. Despite better physical wellbeing, emotional wellbeing (MCS 41.9, SD 11.6) was compromised in comparison with the US female norms and was poorer than has been demonstrated in Australian research in both Rett syndrome [12] (MCS 42.4, SD 10.2) and Down syndrome [11] (MCS 45.2, SD 10.6). In contrast, a large proportion of mothers in the international CDKL5 disorder sample were from the US, and generally younger than those in the Australian studies [11, 12], factors which may also have some bearing on the findings.

Emotional wellbeing of primary care givers was particularly poor when the child was an only child, the child’s co-occurring sleep disturbances were severe, and the family had faced financial hardships to meet the child’s healthcare needs. These associations are broadly consistent with findings of most previous research. Poor maternal sleep quality, often a sequela of child’s sleep disturbances, has been shown to be an important predictor of depression in mothers of children with developmental disabilities, mostly autism spectrum disorder [23–27] or cerebral palsy [28]. Moreover, a similar relationship to that we identified with financial hardship was demonstrated in a Canadian study involving mothers with a child with a developmental disability [29].

Our study reveals some unique findings. Firstly, mental health was least impaired in mothers caring for a child who was totally tube fed, whereas this was the group with the poorest physical health. The literature has shown variability in parent’s experiences for those with a child with a disability after the child’s gastrostomy placement, although enhancement in nutritional status has been reported [30–33]. Parents have reported less burden of care at mealtimes, reduced stress that their child is inadequately nourished, and relief that medications are consistently delivered, consequently improving their emotional wellbeing [30–34]. In a previous study with primary caregivers of a child with Rett syndrome we found that there was general satisfaction with outcomes following gastrostomy insertion [30]. This was related to improved well-being and nutritional status of the child and for the parent reduced care demands and less concern about feeding and the delivery of medication [30]. In this study of the CDKL5 disorder, it is likely that some children may have had the gastrostomy placement in order to facilitate the provision of a ketogenic diet [35], which might have helped control seizures and in turn ease emotional burden of care for the parents. Although primary caregiver’s physical health was not adversely affected by gastrostomy placement in those with a child with cerebral palsy [36], increased demands or excessive weight gain of the child could be contributing to mothers’ physical burden in our study. Thus some deterioration in caregiver physical health may accompany better emotional health, which has been a consequence of less mealtime stress and improved child well-being.

Secondly, while a slight improvement in physical wellbeing was demonstrated, respite care use did not have a positive impact on the mental health of the primary caregivers. Furthermore, families who had utilised these services were less satisfied with every aspect of their family quality of life compared with non-users. Despite contrasting findings from a systematic review [37], individual studies have shown that service utilisation either had no positive effect [29] or was negatively associated with carer’s emotional health [38, 39]. Our longitudinal study involving families with a child with Rett syndrome found that respite care use did not improve parental emotional health and was associated with worse physical health over two years of follow-up [40]. Furthermore, in other research increased family needs for support have been reported to be associated with poorer family quality of life [41]. Our findings might suggest that needs for respite care are not being met for some families and that the current framework for service delivery is not enhancing parental wellbeing or family quality of life. However, this issue warrants further investigation, possibly using either a longitudinal study design and/or qualitiative methods.

Thirdly, mothers in the youngest age group experienced the most impaired emotional health, possibly related to the recency of receiving their child’s diagnosis, a time when they could be experiencing overwhelming fear and loneliness [42]. However, establishing a diagnosis is imperative so that families can clarify their child’s expected life course and gain disease-specific family support [43]. Parents of a child with intellectual disability reported their wellbeing as better when the child had received a definite diagnosis [17]. As the *CDKL5* gene testing has become available over the last decade, some of older primary caregivers in this study might have felt greatly relieved when their adult child eventually received a clear diagnosis. That said even in the general population mental health scores do improve with age [20], but for those with a CDKL5-affected child, we showed that the mean MCS score was consistently poorer across all age groups.

Lastly, family quality of life was strongly mediated by primary caregiver’s health status. The mother’s report of family quality of life has been assessed in most studies [16], and mothers under stress are more likely to consider their family quality of life as poorer [15]. In our study, adjustment for the SF-12v2 scores indicated that child’s sleep difficulty had less impact on the whole family than on the primary carer. Controlling for maternal health would be of use to verify pathways from potential predictors to family quality of life.

We were surprised that there were no strong associations between the frequency of seizures or the attainment of gross motor and communication skills and family outcomes, given the major burden of epilepsy and the severe physical and intellectual impairment in the CDKL5 disorder. Previous studies on the effect of seizure control on maternal mental health have had mixed findings although mostly undertaken in children who were otherwise healthy [44–48]. The marked intellectual disability and various comorbidities among children with the CDKL5 disorder may lessen the influence of seizures alone on the primary caregiver’s health.

Finally, we did not find any association between the rate of hospitalisations and primary caregiver health or family quality of life. In our study, all hospitalisations experienced by the child with the CDKL5 disorder over the life course were included, whereas the outcomes related to current status. We therefore may not be able to observe any true relationships.

Our study has several limitations. First, we used a cross-sectional study design and because of the absence of longitudinal data we were restricted to the reporting of associations rather than causal relationships. Secondly, despite the growth of our international database, the ICDD is not a population-based study. Hence, while remarkable heterogeneity in the carer’s physical health and family quality of life were found according to child’s country of birth, it might be explained by the uneven distribution of participants as well as voluntary participation. Thirdly, we did not account for the child’s age when a diagnosis was made. Time intervals from when parents first had concerns about their child to the age at diagnosis and from the age at diagnosis to the present might influence the primary caregiver’s emotional wellbeing. Fourthly, family quality of life was reported solely by the primary caregiver although adjustment for the SF-12v2 scores would have helped to counteract any confounding effect.

## Conclusions

Despite its shortcomings, we believe that this study provides important insights into primary caregiver’s wellbeing and family quality of life among families with a child with a severe genetic disorder, previously difficult to study because of its rarity. Child’s feeding methods and sleep difficulties, and experiences of financial hardship were associated with primary caregiver’s wellbeing, whereas use of respite care services was the principal correlate with family quality of life after controlling for the carer’s health among families with a child with the CDKL5 disorder included in this study. To date, minimal attention has been given to families with a child living with a rare genetic disorder. Although we still acknowledge the need for further longitudinal investigation, the current research has only been possible for the CDKL5 disorder through the implementation and development of a worldwide database.

## Abbreviations

aa: Amino acid
BCFQOL: Beach center family quality of life
CDKL5: Cyclin-dependent kinase-like 5
ICDD: International CDKL5 disorder database
IFCR: International foundation for CDKL5 research
MCS: Mental component summary
PCS: Physical component summary
RTT: Rett syndrome
SDSC: Sleep disturbance scale for children
SF-12v2: Short form 12 health survey version 2
